# Resveratrol as a protective agent of goat sperm submitted to sex sorting in discontinuous Percoll gradient - preliminary results

**DOI:** 10.1186/1753-6561-8-S4-P149

**Published:** 2014-10-01

**Authors:** Ellen CB Silva, Helder M Souza, Lúcia CP Arruda, Chirlane C Silva, Daniela S Carvalho, Maria Madalena P Guerra

**Affiliations:** 1 Laboratório de Andrologia - Universidade Federal Rural de Pernambuco, Recife, PE, 52171-900, Brazil; 2 Laboratório de Enzimologia - Universidade Federal de Pernambuco, Recife, PE, 50670-901, Brazil

## Background

Sexing sperm is an important biotechnical that enables improve animal production, with achievement of maximum animal breeding. However, all sex semen methodologies can promote irreparable damages to sperm cell, which compromise their fertility [1]. Among the main causes of injures to the sperm are the reactive oxygen species, which have their production intensified during semen manipulation [2]. Therefore, antioxidants therapies can be an important alternative to protect the sperm during laboratory manipulation [3]. Thus, as resveratrol is a powerful antioxidant with an important role on sperm protection [3], the aim of this study was to evaluate the protective effect of resveratrol on goat sperm submitted to sex sorting in discontinuous Percoll gradient.

## Methods

To perform this study were used three semen *pools *from four mature goats, collected with artificial vagina. The fresh semen *pools *(G1) were analyzed and diluted in 10X DMEM to 800 × 10^6 ^sperm/mL. Aliquots of 500 µL of the diluted semen were added on Percoll discontinuous gradients, and prepared according Resende et al. [4], without (G2=sexed semen) or with resveratrol (G3=sexed semen + 75 µM resveratrol). After that, the gradients added of the semen samples were centrifuged (500 × g per 20 min) and the *pellet *of cell recovered. The spermatic cells recovered from each gradient, with or without resveratrol, were evaluated too. The Sperm parameters accessed were plasma membrane integrity (PMi) by CFDA and PI [5], mitochondrial membrane potential (MMP) by JC-1 [5], total motility (TM) and progressive motility (PM) by CASA. Statistical analysis was done with ANOVA and Teste-t at 5% significance.

## Results and conclusions

The percentage of spermatic cells with PMi was higher to G1 (P < 0.05) than to G2 (G1 = 83.00 ± 5.07^a^; G2 = 67.83 ± 0.76^b^; G3 = 76.50 ± 4.82^ab^). No statistics differences (P > 0.05) were observed between all the experimental groups for +MMP (G1 = 87.33 ± 8.31; G2 = 76.50 ± 17.32; G3 = 87.17 ± 9.00), TM (G1 = 84.73 ± 7.12; G2 = 71.50 ± 18.70; G3 = 78.43 ± 7.98) and PM (G1 = 37.35 ± 6.67; G2 = 39.99 ± 12.41; G3 = 48.61 ± 10.12). Nevertheless, sexed semen with resveratrol showed numerically, higher values to the parameters evaluated than without it, fact that consolidates the Sarlós et al. [3] observations about the protective effect of resveratrol. In conclusion, resveratrol does not represent protection to goat sperm submitted to sex sorting in discontinuous Percoll gradient. However, new studies should be realized to reduce the standard variances and determine the real role of resveratrol on sperm protection.
